# C-951-03. Early Oral Opioids Improve Outcomes in the Burn Intensive Care Unit

**DOI:** 10.1093/jbcr/irag033.111

**Published:** 2026-04-06

**Authors:** Jasmine Jin, Artur Manasyan, Sunnie Wong, Haig A Yenikomshian, T Justin Gillenwater, Maxwell B Johnson

**Affiliations:** Keck School of Medicine, University of Southern California, Los Angeles, CA; Los Angeles General Medical Center Regional Burn Center, Los Angeles, CA; Indiana University, Indianapolis, IN; Division of Plastic and Reconstructive Surgery, Keck School of Medicine, University of Southern California, Los Angeles, CA; Division of Plastic and Reconstructive Surgery, Keck School of Medicine, University of Southern California, Los Angeles, CA; Division of Plastic and Reconstructive Surgery, Keck School of Medicine, University of Southern California, Los Angeles, CA

## Abstract

**Introduction:**

In the burn intensive care unit (ICU), pain control is frequently achieved with intravenous opioids (IV). When bolused, these medications have a faster onset but shorter duration of action, which can result in less consistent pain control and greater addictive potential. Our study evaluates whether the early initiation of oral opioids (measured by days until initiation) in the burn intensive care unit (ICU) impacted total narcotic equivalents delivered and important clinical outcomes in early burn care.

**Methods:**

A retrospective review of adult patients admitted to a single burn center from 2015 to 2024 was conducted. Inclusion criteria were age over 18 and total body surface area burned greater than 20%. Exclusion criteria included inpatient mortality, polytrauma, ICU stay less than 72 hours, admission for dermatologic disease, and significant pre-existing comorbidity. Opioids from all sources delivered in the first 14 days of admission were converted to IV morphine equivalents (IME) to standardize the data for comparison. Outcomes included total IME (oral and IV), total number of days on parenteral opioids, total ICU days, and total ventilator days. Statistical analysis included multivariate linear regression to assess the association between the number of days until enteral opioid administration and these outcomes.

**Results:**

A total of 192 patients with a mean age of 43.5 ± 14.3 were included in this study. The cohort had a mean ICU length of stay of 25.1 ± 28.3 days and a mean duration of mechanical ventilation of 13.3 ± 24.2 days. On average, oral opioids were initiated 2.9 ± 3.3 days after admission, with 18.8% of patients not receiving oral opioids at all in the first 14 days. The total IME across the first 14 days was 1150.6 ± 1041.0. Multivariate linear regression analysis controlling for TBSA showed that there was a statistically significant association between later oral opioid initiation and increased total IME use (Coeff 91.692, *p*<.005), longer ICU stays (Coeff 1.256, *p*<.005), and more ventilator days (Coeff 0.941, *p*<.005).

**Conclusions:**

Patients who were started on oral opioids earlier in their treatment of burn injuries showed improved outcomes, including decreased total opioid use, shorter length of ICU stay, and less time on a mechanical ventilator. Although confounding factors like severity of injury and severity of critical illness could impact this relationship, our findings suggest that early initiation of enteral opioids may benefit critically ill burn patients.

**Applicability of Research to Practice:**

The early initiation of oral opioids in burn patients is associated with improved clinical outcomes. These findings support consideration of early enteral opioid therapy, even in patients anticipated to require prolonged sedation or aggressive pain regimens.

**Funding for the study:**

N/A.

